# Primary Intraparenchymal Squamous Cell Carcinoma of the Kidney: A Rare and Unique Entity

**DOI:** 10.1155/2014/256813

**Published:** 2014-01-20

**Authors:** Prithwijit Ghosh, Kaushik Saha

**Affiliations:** ^1^DESUN (NEON) Reference Lab, Kolkata, West Bengal, India; ^2^Department of Pathology, Murshidabad Medical College and Hospital, Berhampore, Murshidabad, West Bengal, India

## Abstract

Primary squamous cell carcinoma (SCC) of the renal parenchyma is a very unusual entity which needs to be differentiated from primary SCC of renal pelvis, SCC from another primary site, and urothelial carcinoma with extensive squamous differentiation. We are most probably describing the second case of primary SCC of the renal parenchyma in a 51-year-old male who presented with heaviness of right upper abdomen with intermittent pain in right flank. Contrast-enhanced computed tomography (CECT) revealed a mass in the right lower pole of the kidney and histopathology following nephrectomy displayed the features of well-differentiated squamous cell carcinoma without urothelial involvement.

## 1. Introduction

Primary squamous cell carcinoma (SCC) of the renal pelvis is a rare but relatively known entity representing only about 0.5 to 15% of all the urothelial cancers. It is often unsuspected clinically due to its rarity and inconclusive clinical and radiological features, and hence patients present at advanced stages resulting in poor prognosis [1]. However, extensive review of the literature reveals only a single case of primary SCC of renal parenchyma reported till date [2]. We probably report the second case of primary SCC of renal parenchyma in a 51-year-old man presenting with nonspecific clinical complaints.

## 2. Case Report

**Figure 1:**
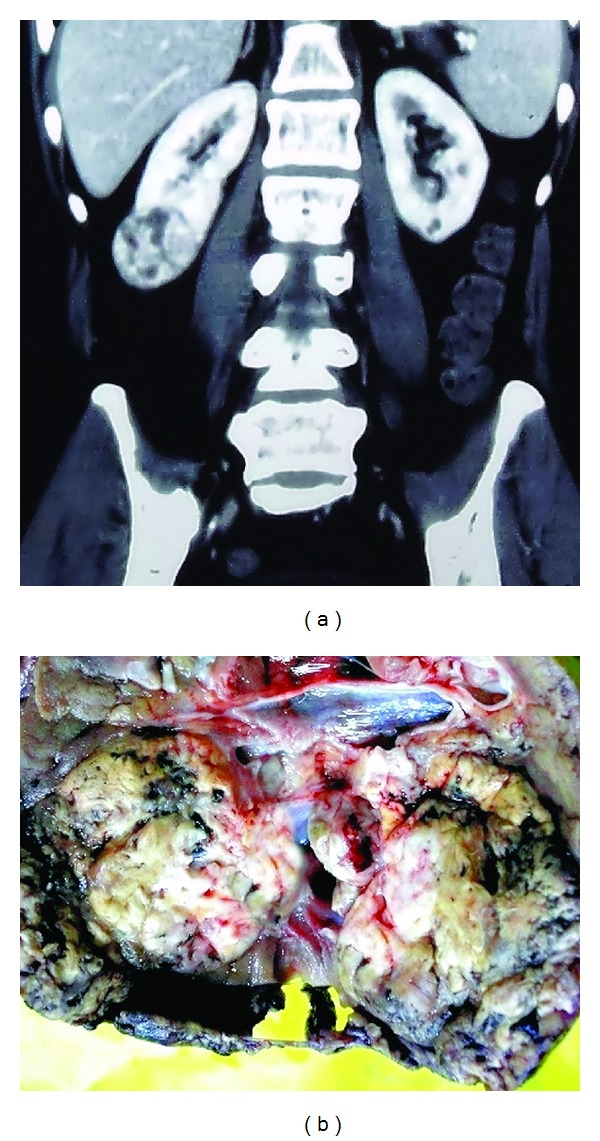
(a) CECT of abdomen on coronal plane showing a solitary mass in the lower pole of right kidney. (b) Photograph of bisected specimen of nephrectomy showing a well-delineated mass in the lower pole.

**Figure 2:**
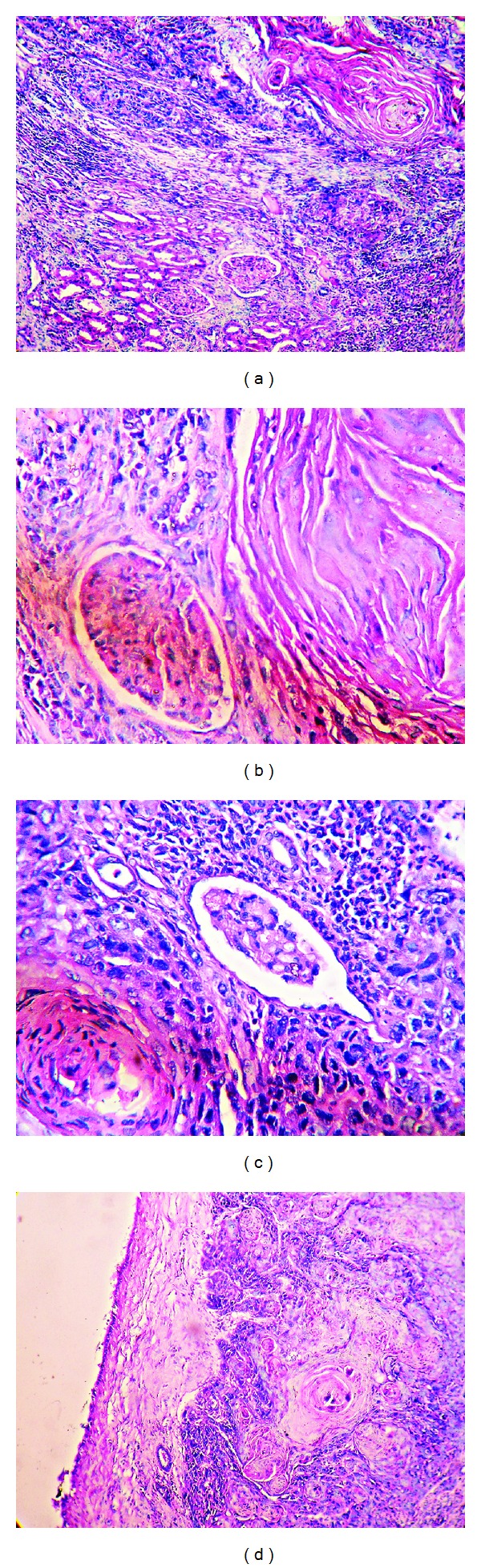
(a) Photomicrograph of well-differentiated squamous cell carcinoma with keratin pearl formation along with glomeruli and tubules (H and E, ×100). (b) Glomerulus and tubules in close relation to keratin pearl of squamous cell carcinoma (H and E, ×400). (c) Photomicrograph of entrapped glomerulus and renal tubules within squamous cell carcinoma (H and E, ×400). (d) Photomicrograph of uninvolved flattened urothelium of pelvicalyceal system (left) keeping a distance from sheets of malignant squamous cells (right) (H and E, ×100).

A 51-year-old male presented with heaviness of right upper abdomen for last 8 months and dull and intermittent pain in the right flank, off and on for last five months. There was no history of weight loss and hematuria during this period. History of fever with associated urinary complaints was also conspicuously absent. He was a nonsmoker and nonhypertensive. The clinical examination revealed mild pallor and mild tenderness in the right flank. There was no palpable lymph node. On routine hematological investigation, his hemoglobin level was 10.2 g/dL and RBCs displayed normocytic normochromic features on peripheral blood film examination. The erythrocyte sedimentation rate (ESR) was 40 mm after the 1st hour. Serum urea and creatinine values were within normal limits. Urine analysis revealed mild pyuria which was sterile on culture. Urine dip-stick test was negative for blood and urinary RBC count was within normal limit. However, mild proteinuria was detected. A solitary heterogeneously enhancing relatively well-delineated mass situated in the lower pole of right kidney was detected on contrast-enhanced computed tomography (CECT) scan without any noticeable infiltration of adjacent organs (Figure 1(a)). Retroperitoneal lymph nodes did not appear to be enlarged on CECT. There was no feature of associated hydronephrosis or calculi. Further, no distant metastases were appreciated on CECT chest or bone scan. He underwent a right total nephrectomy without any complication. On gross examination, the mass was variegated, light tan to yellow, friable (Figure 1(b)) measuring 5.8 cm × 5.5 cm × 4.5 cm confined to the lower pole with cut section revealing areas of hemorrhage and necrosis. The mass did not grossly involve the pelvicalyceal system. There was no calculus or significant cystic dilatation of renal pelvis. Histopathology displayed the features of well-differentiated squamous cell carcinoma with nests of large atypical squamous epithelial cells, keratin pearl formation, and focal areas of necrosis in the renal parenchyma with entrapped glomeruli and tubules (Figures 2(a), 2(b), and 2(c)). The surrounding areas showed a chronic inflammatory reaction. Renal vein, perinephric tissue, and Gerota's fascia remained uninvolved (TNM stage T1bN0M0). Intra- or peritumoral lymphovascular invasion was not detected. Meticulous sampling of the pelvicalyceal system revealed that the nearest urothelium was absolutely free from the tumour mass and did not harbor any feature of squamous metaplasia and of squamous carcinoma in situ (Figure 2(d)). An 18-Fludeoxyglucose positron emission tomography/computed tomography (FDG-PET/CT) scan failed to demonstrate any other unknown primary site. The patient did not receive any adjuvant therapy and was alive and doing well after 6 and 12 months of surgical resection with no evidence of recurrence or metastasis.

## 3. Discussion

Transitional cell carcinoma is reportedly the most common type originating in the renal pelvis followed by SCC which is relatively rare and affects predominantly women in the age group of 50 to 70 years. However, SCC of the renal pelvis usually presents at an advanced stage with infiltration of adjacent tissue though both usually tend to have similar prognosis at later stages [3]. In the present case, the tumor was a primary renal intraparenchymal SCC detected in a male patient at an earlier stage with excellent post treatment outcome.

SCC of the urothelial tract is thought to arise through a process of metaplasia mostly keratinizing squamous metaplasia of the urothelium which increases the chances of squamous cell carcinoma in future. The concept of squamous metaplasia as the forerunner of SCC of urothelial tract however has been inflicted with controversies, with differing results from previous studies. The disagreement may partially be due to the relative rarity of SCC of the upper urinary tract [4]. In the presence of an identifiable urothelial dysplastic element including urothelial CIS (carcinoma in situ), the tumor should be classified as primary urothelial carcinoma with squamous differentiation. However, the conspicuous presence of keratinizing squamous metaplasia of the adjacent flattened urothelium, especially if associated with dysplasia, supports a diagnosis of primary SCC of the renal pelvis which is rare [5, 6]. No such dysplastic urothelial component or metaplastic and/or dysplastic squamous lining of urothelium was found in this case.

The etiological factors, namely, recurrent urinary tract infections with or without vesicoureteric reflux, long standing staghorn calculi, smoking, schistosomiasis, exogenous and endogenous chemicals, vitamin A deficiency, hormonal imbalance, and so forth, are the leading ones in renal pelvis SCC. Only few cases have been reported where no apparent aetiological factor could be detected [4, 7]. Our case notably lacked the association with such predisposing factors.

Primary SCC of the kidney should further be distinguished from metastatic SCC with the combination of clinical history, imaging studies, and histopathology [4, 8].

After ruling out the metastasis, for it to be stamped as a primary renal parenchymal SCC, an extremely rare entity, most importantly the renal pelvis, should be histologically normal, besides other findings as in our case. In the present case, the above-mentioned aetiological factors were absent, CT scan revealed a solitary renal mass, PET scan failed to detect any other unknown primary sites, and histopathological evidence of squamous metaplasia or dysplasia of the urothelium was totally absent despite extensive sampling of the pelvicalyceal region. All these together confirmed the primary intraparenchymal origin of SCC of the kidney. Thus our case shows the need for the consideration of this rare entity in differential diagnosis of renal SCC. As the prevailing data regarding the incidence, histogenesis, disease course, and prognosis of primary intraparenchymal SCC are very inadequate, it needs further future evaluation to provide comprehensive data on this entity.
